# Association between U.S. State AIDS Drug Assistance Program (ADAP) Features and HIV Antiretroviral Therapy Initiation, 2001–2009

**DOI:** 10.1371/journal.pone.0078952

**Published:** 2013-11-18

**Authors:** David B. Hanna, Kate Buchacz, Kelly A. Gebo, Nancy A. Hessol, Michael A. Horberg, Lisa P. Jacobson, Gregory D. Kirk, Mari M. Kitahata, P. Todd Korthuis, Richard D. Moore, Sonia Napravnik, Pragna Patel, Michael J. Silverberg, Timothy R. Sterling, James H. Willig, Ann Collier, Hasina Samji, Jennifer E. Thorne, Keri N. Althoff, Jeffrey N. Martin, Benigno Rodriguez, Elizabeth A. Stuart, Stephen J. Gange, for the North American AIDS Cohort Collaboration on Research and Design (NA-ACCORD) of the International Epidemiologic Databases to Evaluate AIDS (IeDEA)

**Affiliations:** 1 Johns Hopkins University, Baltimore, Maryland, United States of America; 2 Albert Einstein College of Medicine, Bronx, New York, United States of America; 3 Centers for Disease Control and Prevention, Atlanta, Georgia, United States of America; 4 University of California San Francisco, San Francisco, California, United States of America; 5 Mid-Atlantic Permanente Research Institute, Rockville, Maryland, United States of America; 6 University of Washington, Seattle, Washington, United States of America; 7 Oregon Health and Science University, Portland, Oregon, United States of America; 8 University of North Carolina at Chapel Hill, Chapel Hill, North Carolina, United States of America; 9 Kaiser Permanente Northern California, Oakland, California, United States of America; 10 Vanderbilt University, Nashville, Tennessee, United States of America; 11 University of Alabama at Birmingham, Birmingham, Alabama, United States of America; 12 British Columbia Centre for Excellence in HIV/AIDS, Vancouver, British Columbia, Canada; 13 Case Western Reserve University, Cleveland, Ohio, United States of America; Infectious Disease Service, United States of America

## Abstract

**Background:**

U.S. state AIDS Drug Assistance Programs (ADAPs) are federally funded to provide antiretroviral therapy (ART) as the payer of last resort to eligible persons with HIV infection. States differ regarding their financial contributions to and ways of implementing these programs, and it remains unclear how this interstate variability affects HIV treatment outcomes.

**Methods:**

We analyzed data from HIV-infected individuals who were clinically-eligible for ART between 2001 and 2009 (i.e., a first reported CD4+ <350 cells/uL or AIDS-defining illness) from 14 U.S. cohorts of the North American AIDS Cohort Collaboration on Research and Design (NA-ACCORD). Using propensity score matching and Cox regression, we assessed ART initiation (within 6 months following eligibility) and virologic suppression (within 1 year) based on differences in two state ADAP features: the amount of state funding in annual ADAP budgets and the implementation of waiting lists. We performed an *a priori* subgroup analysis in persons with a history of injection drug use (IDU).

**Results:**

Among 8,874 persons, 56% initiated ART within six months following eligibility. Persons living in states with no additional state contribution to the ADAP budget initiated ART on a less timely basis (hazard ratio [HR] 0.73, 95% CI 0.60–0.88). Living in a state with an ADAP waiting list was not associated with less timely initiation (HR 1.12, 95% CI 0.87–1.45). Neither additional state contributions nor waiting lists were significantly associated with virologic suppression. Persons with an IDU history initiated ART on a less timely basis (HR 0.67, 95% CI 0.47–0.95).

**Conclusions:**

We found that living in states that did not contribute additionally to the ADAP budget was associated with delayed ART initiation when treatment was clinically indicated. Given the changing healthcare environment, continued assessment of the role of ADAPs and their features that facilitate prompt treatment is needed.

## Introduction

Reducing HIV-related health disparities is a priority of the United States (U.S.) National HIV/AIDS Strategy (NHAS) [1]. Many U.S. studies have demonstrated marked disparities in HIV health care use and outcomes by factors such as race/ethnicity [2], insurance status [3], and transmission risk [4], [5]. For example, people with HIV infection who use illicit drugs have been found to be less likely to receive antiretroviral therapy (ART) [6], [7], although gaps have been decreasing in more recent years [8]/ Furthermore, geographic variation has been linked with differences in treatment initiation [7], [9], hospitalizations [10], [11], and mortality [12] in HIV-infected people. State policy differences likely contribute to geographic disparities; individuals infected with HIV are often dependent on public health care services [13], whose guiding policies are largely determined at the state level.

In particular, differences by state response to the Ryan White CARE Act Part B AIDS Drug Assistance Programs (ADAPs), which are used by about one-quarter of HIV-infected individuals in care in the United States [13], may affect the timeliness of obtaining treatment, as well as the benefits of such treatment. State ADAPs act as the “payer of last resort” in providing ART and other prescription medications to eligible people with HIV infection [14]. People are eligible for ADAP services if they do not have their own prescription drug coverage and do not qualify for coverage through Medicare or their own state's Medicaid program (i.e., the inadequately insured, the less sick, and/or the working poor). While ADAPs receive federal funding annually through the Ryan White HIV/AIDS Program, each state administers its program independently. As a result, ADAPs differ in many ways, including the additional criteria used to define who is eligible for ADAP assistance, the comprehensiveness of the state ADAP drug formulary, and the procurement of additional funding by the ADAP through sources such as state general revenue [14]. This last factor is relevant because federal allocations may not cover the full needs of a state, and therefore many states supplement the ADAP budget using monies from state funds, which in Fiscal Year 2011 made up 16% of the national ADAP budget [15]. Additionally, some state ADAPs over the years have instituted enrollment waiting lists, an action that has been particularly scrutinized, since these lists may delay people from receiving ART, which in turn prevents them from benefiting clinically from timely ART [16], [17]. Waiting lists reached peak use in 2011, when 14 states had an active waiting list, representing 9,298 people who had applied for ADAP services but were not yet able to access medications through their states' programs [18].

The published research on the clinical consequences of specific features of ADAPs, primarily based on mathematical modeling, has found the overall program to be cost-effective [19], and that more generous state ADAPs are associated with better health outcomes, including a lower incidence of opportunistic illnesses and lower mortality [20]–[22]. Empirical data from observational studies offer an opportunity to corroborate these findings and better understand potential barriers to ADAP enrollment and therefore timely initiation of treatment. Such information is important as states manage their programs under increasingly greater client demand and limited resources [18], [23].

To understand the association between state ADAP policies and treatment outcomes, we assessed differences in ART initiation and viral load suppression among newly treatment-eligible participants in U.S. cohorts of the North American AIDS Cohort Collaboration on Research and Design (NA-ACCORD), a collaboration of prospective cohort studies of HIV-infected individuals in the U.S. and Canada, between 2001 and 2009. We compared these outcomes based on two potentially unfavorable ADAP circumstances: not having additional state funding in the annual ADAP budget and the use of waiting lists. Our research question was whether individuals living in states under each of these circumstances were less likely to have timely ART initiation and virologic suppression, compared with similar individuals not living in states under the same circumstances. A secondary question was whether these differences were more pronounced among those with a history of injection drug use. We hypothesized that effects would be greater in this population, owing to their greater needs with respect to engagement in care and starting treatment [24], [25].

## Methods

### Data source and study population

NA-ACCORD is a collaboration of single- and multi-site HIV cohorts that includes over 100,000 individuals from more than 100 research sites in the U.S. and Canada [26]. At least annually, each participating NA-ACCORD cohort submits standardized data regarding enrolled participants' demographic characteristics, prescribed antiretroviral therapies, laboratory tests, clinical diagnoses, and vital status to a centralized Data Management Core, where the data undergo quality control for completeness and accuracy before being combined into harmonized analysis files.

The source population for our analyses consisted of HIV-infected individuals in the NA-ACCORD who were newly eligible to initiate ART between 2001 and 2009, based on existing treatment guidelines during this period (an incident AIDS-defining event or CD4+ lymphocyte [CD4+] count recorded <350 cells/uL) [27] from 14 U.S. cohorts. Inclusion criteria included known residence within a U.S. state, no prior CD4+ counts <350 cells/uL or AIDS-defining illnesses documented, at least two CD4+ counts in the study period, and no prior use of ART documented.

Because we were interested in answering the question of whether individuals would have had different outcomes if they did not live in a state without a particular ADAP characteristic, we limited certain analyses to a subset of individuals who lived in states with that particular feature in place at the time of ART eligibility, and similar individuals who lived in states without that feature.

In a secondary analysis, we examined individuals with a documented history of injection drug use (IDU). To account for potential underreporting of IDU, we also included individuals without a documented history of IDU but with a diagnosis of hepatitis C infection recorded in the absence of either a report of hemophilia, contact with blood products, or among men, sex with men. While this may have included some individuals without a history of IDU, we conducted sensitivity analyses excluding these additional individuals.

*Ethics*: The activities of the NA-ACCORD have been reviewed and approved by the local institutional review boards (IRBs) for each site. This study was determined to not qualify as human subjects research by the Johns Hopkins Bloomberg School of Public Health IRB.

### Outcomes of interest

Our first outcome of interest was time to ART initiation, using the date of ART eligibility (i.e., the first date that an incident AIDS-defining illness or a CD4+ count <350 cells/uL was recorded) as the time origin. Time to ART initiation was defined as the duration between the date of eligibility and the date an ART regimen was prescribed (denoted in the medical record), or if this was not available, when a regimen was started (denoted by self-report). Time was censored at six months after eligibility to focus on more timely treatment initiation. ART regimens comprised at least three active antiretroviral agents, including a protease inhibitor, a non-nucleoside reverse transcriptase inhibitor, an entry inhibitor, or an integrase strand transfer inhibitor; or three nucleoside reverse transcriptase inhibitors, including abacavir or tenofovir. Ritonavir in the presence of another protease inhibitor was not included in this definition.

The other outcome of interest was time from ART eligibility to viral load (VL) suppression (within one year). Suppression was based on a laboratory result report of an HIV-1 RNA level ≤500 copies/mL. This threshold was used to account for differences in detection limits of commercial assays over the study period [28].

### Variables of interest

For each individual in our study, the two state ADAP features in place on the date of ART eligibility were assessed and stratified into dichotomous categories that could be classified as more cost-containing versus less cost-containing: (1) amount of state funding provided to the annual ADAP budget (none vs. any); and (2) use of waiting lists in the state (yes vs. no). Information on state ADAP features was derived from the results of surveys conducted by the National Alliance of State and Territorial AIDS Directors (NASTAD) and published in annual reports [15]. We initially considered two additional state ADAP features, financial eligibility criteria for ADAP enrollment and inclusiveness of the state ADAP drug formulary with respect to commercially available antiretroviral drugs, but found limited variation in these variables across states (Table 1) (e.g., most states have a comprehensive ART formulary), restricting our ability to assess their impact on the outcomes of interest. State-level ADAP variables were linked to individuals by their state of residence at the time of ART eligibility (i.e., these values varied by time). For three multi-site cohorts, the state of residence was not available, and the state of the clinic site was used instead as a proxy. We hypothesized that living in a state with a less generous ADAP feature would be associated with delayed ART initiation and virologic suppression (i.e., a hazard ratio less than one).

**Table 1:** Comparison of demographic characteristics and AIDS Drug Assistance Program features of U.S. states represented in study, 2001 and 2009 data.

Characteristic	2001				2009			
	All U.S. states*		34 states* in study		All U.S. states*		34 states* in study	
	Median	IQR	Median	IQR	Median	IQR	Median	IQR
Demographic variables								
Population density (per square mile)	90	42–221	137	63–274	100	43–230	150	66–282
% of population that is of black race	7.2	2.3–15.8	10.9	4.1–19.8	7.6	3.1–16.3	11.5	5.3–19.7
Annual household income (current U.S. dollars, thousands)	51,004	46,473–58,205	51,663	47,095–56,861	49,909	45,455–56,568	49,271	45,036–56,853
% of population living below FPL	10.5	8.5–14.1	11.1	8.5–14.2	13.3	10.9–15.8	13.9	11.7–16.6
State Medicaid HIV spending per capita	N/A	N/A	N/A	N/A	18,757	15,768–22,710	19,621	16,417–23,088
AIDS Drug Assistance Program (ADAP) features								
% state contribution to total ADAP budget expenditures	9	0–21	14	3–28	11	0–25	19	5–31
States contributing to total ADAP budget, by percentage (N, %)								
0%	N = 15	29%	N = 6	18%	N = 17	33%	N = 8	24%
Less than 20%	N = 22	43%	N = 17	50%	N = 15	29	N = 9	26%
20% or more	N = 14	27%	N = 11	32%	N = 19	37%	N = 17	50%
% of all available antiretroviral drugs on formulary	100	100–100	100	100–100	100	97–100	100	97–100
Financial eligibility threshold as % of FPL	300	230–350	300	281–370	300	300–400	300	300–400
States with waiting list at least once during study (N, %)	-	-	-	-	N = 20	39%	N = 11	32%

* Including the District of Columbia.

FPL = federal poverty level, IQR = interquartile range, N/A = not available. State demographic variables from annual U.S. Census population estimates and the Current Population Survey [31], [33]. State Medicaid spending from the Kaiser Commission on Medicaid and the Uninsured and the Urban Institute [32]. ADAP features from the National Alliance of State and Territorial AIDS Directors (NASTAD) [15].

### Other variables

Other individual-level variables assessed at the time of ART eligibility and included as potential confounders were age, race/ethnicity (black; Hispanic; white or other), sex and transmission risk (men who have sex with men; male IDU; female IDU; male heterosexual or other risk, female heterosexual or other risk), CD4+ count, HIV viral load, calendar year, and documented histories of drug abuse, alcohol abuse, and mental illness. Drug abuse, alcohol abuse, and mental illness were categorized on the basis of more specific diagnoses derived from electronic medical record diagnoses and chart reviews. As potential psychosocial barriers to ART initiation, they were grouped as a single ordinal variable, representing the number of barriers experienced [29].

To account for differences in ART initiation influenced by characteristics of the cohorts or clinics themselves, we categorized cohorts into the following categories: multi-site clinical cohort, single-site clinical cohort, and interval cohort. Interval cohorts differ from clinical cohorts in both timing and data collection; individuals are followed at specified intervals (e.g., every six months) that are unrelated to health care visits, and data are collected according to defined protocols [30]. We also included two variables representing specific mechanisms undertaken by individual clinics to assist with access to ART drugs. This information came from the results of a standardized questionnaire given in September 2011 to all clinical cohorts contributing data to this study. Mechanisms were divided into those performed by clinic staff and those done after referral to entities outside the clinic. Additional information about the questionnaire is in File S1.

State-specific characteristics related to population demographics and Medicaid spending may also affect decisions on how ADAPs are run, as well as ART initiation. To account for these potential confounding differences, we included the following state variables, linked to individuals by the year of ART eligibility and categorized into quartiles: population density [31], the percentage of the population who are of black race, the percentage of the population living below the federal poverty line, median household income, and per capita Medicaid spending on enrollees with HIV. Medicaid data came from the Henry J. Kaiser Family Foundation and were available for the years 2007–2009 only [32]. All other data were available from all years (i.e., these values varied by time) and came from annual U.S. Census population estimates [31] and the Current Population Survey [33].

### Statistical methods

To estimate the effect of each ADAP characteristic on treatment outcomes, we used propensity score matching to account for potential differences between persons living in a state with a specific ADAP characteristic (“exposed” participants) and persons living in a state without that characteristic (“unexposed” participants). Details of the use of this method are included in File S1. Briefly, for each characteristic, we developed a multivariable logistic regression model to estimate the predicted probability of living in a state with that feature, controlling for individual- and clinic-level variables that might confound the relationship between the exposure and the outcomes of interest. We then matched exposed participants to comparable unexposed participants based on the propensity of exposure, using 1∶3 nearest neighbor matching (i.e., matching to the unexposed participant with the most similar propensity score), with replacement. Balance on potential confounders between exposed and unexposed participants was evaluated quantitatively and graphically. Propensity score matching was performed using the MatchIt package [34] in R 2.15.0 (R Foundation for Statistical Computing, Vienna, Austria). After matching, we used Cox regression to examine differences in the time to ART initiation and time to VL suppression by each ADAP feature. Models were adjusted for the propensity score and any additional variables with residual imbalance, and weighted to account for matching with replacement. The resulting inferences are generalizable to persons who are similar to those living in states with the less generous ADAP characteristic, maximizing internal validity in this subset of individuals [35].

We also performed analyses that did not use propensity score matching but rather conventional multivariable Cox regression analysis. Such models may be less able to adjust for known confounders if there is limited covariate overlap, but use the entire study population instead of a more limited subset. We also used conventional Cox regression analysis for our pre-specified subgroup analysis among IDU, because we could not get adequate balance on confounders in the propensity score model.

To further explore the relationship between a state contribution to the annual ADAP budget and increases in ART initiation, we looked for evidence of a “dose-response” trend in state funding. Because our propensity score models used logistic regression and thus require a dichotomous “treatment”, we used conventional Cox models to explore this relationship. We created three levels of state funding: 0% of the total ADAP budget (i.e., no state contribution), >0% but <20%, and 20% or more.

Finally, we performed several sensitivity analyses to examine assumptions about the relationship between state ADAP characteristics and the outcomes of interest. These included use of alternate statistical methods, modifications to the exposure definition, and additional subgroup analyses (see File S1 for details).

## Results

**Figure 1:**
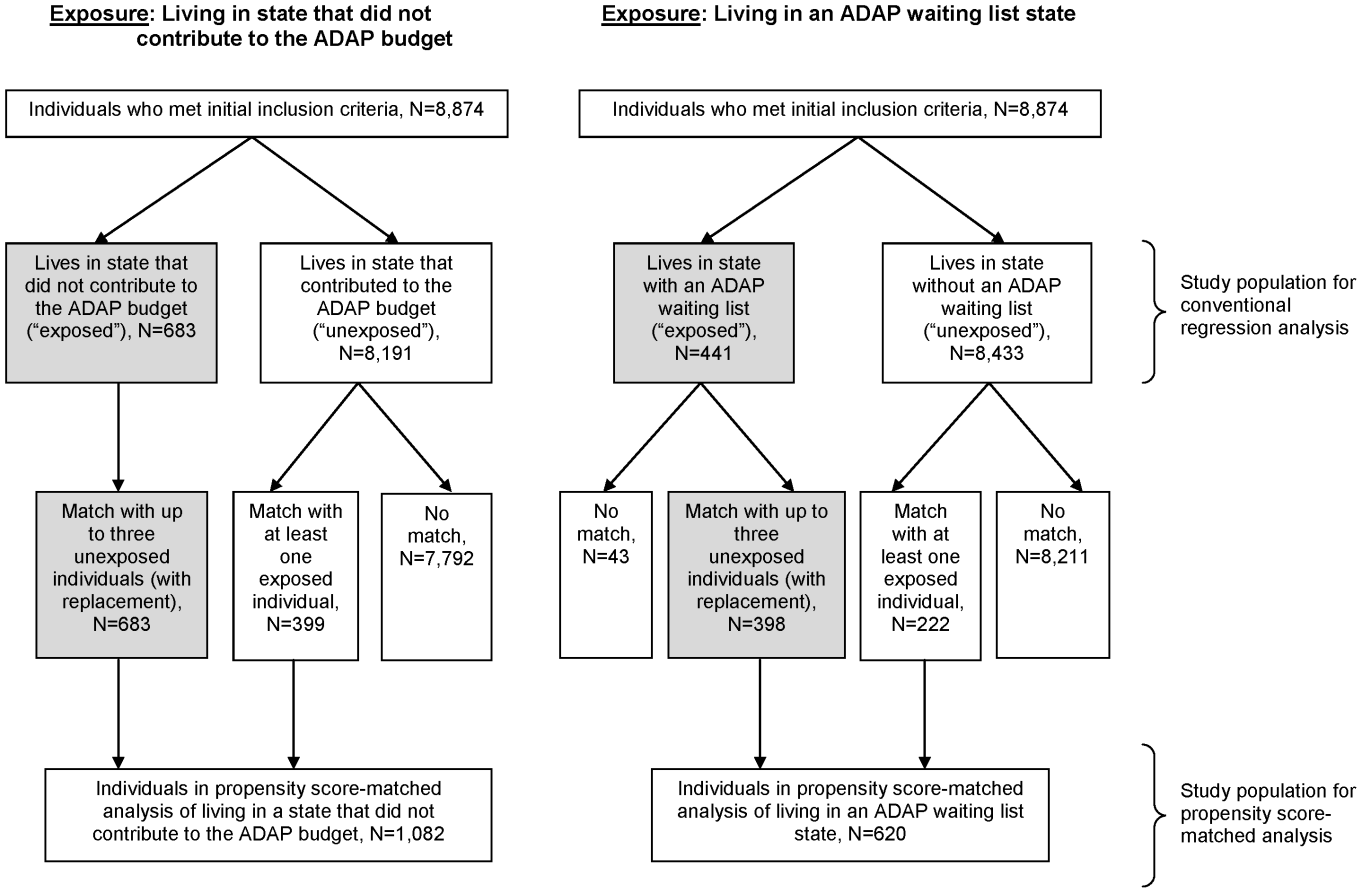
Flow charts showing selection into each of the two analyses. Gray indicates the population of interest for the propensity score-matched analyses.

**Figure 2:**
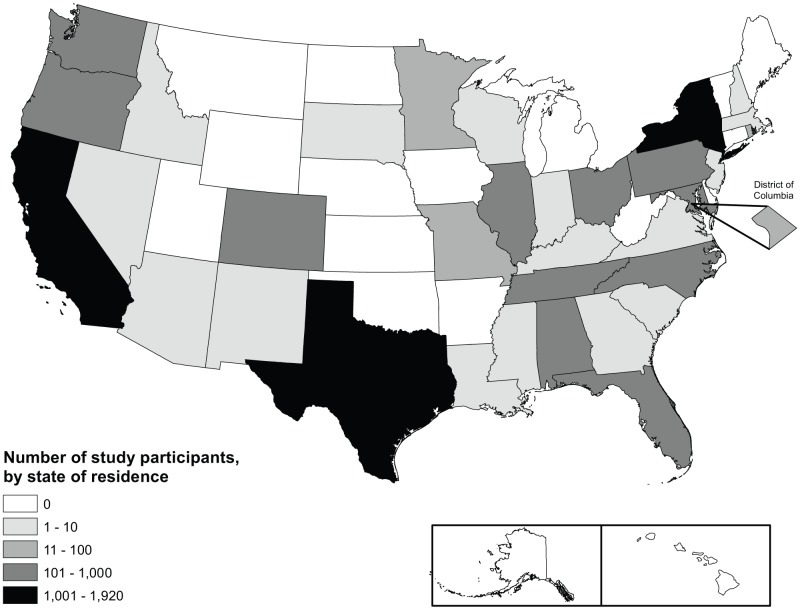
Map of U.S. states represented in study.

There were 8,874 individuals initially eligible between 2001 and 2009 for inclusion in this analysis. Figure 1 shows the study selection process used to identify individuals living in states with the ADAP characteristics under question, and similar individuals not living in these states, used in propensity score analyses. Overall, the median age was 40 years, and 74% were men (Table 2). Among men, 59% reported sex with men as a transmission risk factor, 14% reported IDU and 27% reported heterosexual transmission or other risk. Among women, 17% reported IDU and 83% reported heterosexual or other risk. Among all individuals, 44% were black, 33% were white, 18% were Hispanic, and 4% were Asian or of other race/ethnicity. The overall study population lived in 33 states and the District of Columbia (Figure 2).

**Table 2:** Characteristics of newly treatment-eligible HIV-infected U.S. residents in NA-ACCORD, 2001–2009.

	Overall (N = 8,874)		Included in analysis of state contribution to ADAP budget (N = 1,082) *		Included in analysis of state ADAP waiting lists (N = 620) *	
	N	%	N	%	N	%
Age at eligibility, years (median, IQR)	40	33–46	41	34–47	37	31–44
18–29	1,555	18	139	13	131	21
30–39	2,869	32	343	32	236	38
40–49	2,989	34	397	37	196	32
50–59	1,216	14	181	17	47	8
60+	245	3	22	2	10	1.6
Race/ethnicity						
Black (non-Hispanic)	3,937	44	617	57	272	44
Hispanic	1,631	18	57	5	40	7
White (non-Hispanic)	2,944	33	382	35	293	47
Other (non-Hispanic)	362	4	26	2.4	15	2.4
Sex and transmission risk						
Men who have sex with men	3,839	43	368	34	282	46
Male injection drug user	946	11	210	19	46	7
Male, heterosexual or other risk	1,764	20	162	15	145	23
Female injection drug user	387	4	115	11	12	1.9
Female, heterosexual or other risk	1,938	22	227	21	135	22
Eligibility criteria						
CD4+ count 0–199 cell/uL	3,118	35	274	25	224	36
CD4+ count 200–349 cells/uL	5,464	62	775	72	380	61
Incident AIDS-defining illness (i.e., CD4+ count not <350 cells/uL)	292	3	33	3	16	2.6
Viral load at eligibility						
501–999 copies/mL	152	1.7	12	1.1	6	1
1,000–9,999 copies/mL	1,299	15	156	14	56	9
10,000–99,999 copies/mL	3,743	42	464	43	248	40
100,000+ copies/mL	2,588	29	261	24	162	26
Missing	1,092	12	189	18	148	24

ART = antiretroviral therapy, IQR = interquartile range. Percentages may not add up to 100 due to rounding.

* See Figure 1 for details of study selection procedure.

In Table 1, we show state-level demographic and socioeconomic characteristics based on 2001 and 2009 data of the 34 jurisdictions represented by the overall study population, as well as a comparison to the U.S. overall. States in this study were more densely populated, more diverse with respect to black race, had a greater percentage of their population living below the federal poverty line, and spent more Medicaid dollars on HIV per capita. Table 1 also shows the distribution of selected ADAP features in these states. The percent state contribution to the state's total ADAP budget was not significantly associated with having an ADAP waiting list.

Regarding the mechanisms undertaken by individual clinics to assist with access to ART drugs (Table S1 in File S1), clinics on average had four procedures in place to directly assist their patients with accessing prescription drugs, including assisting patients with ADAP enrollment (91%), Medicare Part D, and Medicaid (both 86%), and pharmaceutical assistance programs (77%). 64% also had mechanisms in place to refer patients to other organizations for additional help.

### Association between no state ADAP contribution and treatment outcomes

In the overall study population (N = 8,874), 56% of individuals initiated ART within six months of eligibility. Persons living in states not contributing to the ADAP budget were less likely to initiate ART within six months than persons living in states that did (39% vs. 58%). Table 3 shows crude and adjusted conventional Cox regression-based hazard ratios for the association between living in a state contributing to its ADAP budget and ART initiation (adjusted hazard ratio [HR] 0.80 [95% CI 0.69–0.93]). After propensity score matching, the association between living in a state with no additional state contribution to the ADAP budget and delayed ART initiation retained statistical significance (N = 1,082, HR 0.73, 95% CI 0.60–0.88). We also found a significant dose-response relationship: compared with living in a state with a 20% or greater state contribution, the HR for ART initiation when living in a state with more than 0% but less than <20% contribution was 0.90 (95% CI 0.82–0.99), and the HR for no contribution was 0.75 (95% CI 0.63–0.88) (p_trend_<0.001). In the analysis limited to IDU, the adjusted hazard ratio for ART initiation was 0.67 (95% CI 0.47–0.95), and the dose-response effect persisted. Other sensitivity analyses examining alternative approaches or within different subgroups showed generally consistent findings with the base case results, although some of these associations did not reach statistical significance (Table S2 of File S1).

**Table 3:** Association between living in a state not contributing to the annual ADAP budget and ART initiation and virologic suppression, U.S. NA-ACCORD, 2001–2009.

	Outcome: 6-month ART initiation		Outcome: 1-year virologic suppression	
	HR	95% CI	HR	95% CI
Overall				
No contribution (vs. any contribution)				
Crude (N = 8,874)	0.56	0.49–0.63	0.75	0.67–0.83
**Regression-adjusted (N = 8,874)**	**0.80**	**0.69–0.93**	**1.02**	**0.88–1.18**
**Propensity score-matched (N = 1,082)** *	**0.73**	**0.60–0.88**	**1.13**	**0.93–1.36**
Dose-response effect (P_trend_) (N = 8,874)		<0.001		0.25
No contribution	0.75	0.63–0.88	1.06	0.91–1.24
Contribution <20%	0.90	0.82–0.99	1.07	0.97–1.17
Contribution >20%	1.00	Ref.	1.00	Ref.
Injection drug users only (N = 1,824)				
No contribution (vs. any contribution)				
Crude	0.40	0.31–0.51	0.78	0.64–0.96
**Regression-adjusted**	**0.67**	**0.47–0.95**	**1.14**	**0.82–1.59**
Dose-response effect (P_trend_)		0.005		0.29
No contribution	0.58	0.40–0.86	1.21	0.83–1.74
Contribution <20%	0.81	0.63–1.04	1.10	0.85–1.42
Contribution >20%	1.00	Ref.	1.00	Ref.

ART = antiretroviral therapy, CI = confidence interval, HR = hazard ratio.

All analyses use Cox proportional hazards regression.

* Hazard ratios obtained after 1∶3 matching (with replacement) 683 “exposed” to 399 “unexposed” individuals based on propensity of living in a state contributing to the ADAP budget.

Both regression-adjusted and propensity-score matched analyses account for the following variables: age; sex; race/ethnicity; transmission risk; CD4+ count and viral load at eligibility; history of alcohol abuse, substance abuse, and mental disorders; year of eligibility; type of cohort; clinic-specific mechanisms to help obtain ART; state-level population density, % population of black race, % population below poverty line, median household income, and per capita Medicaid spending on HIV.

Virologic suppression one year after ART eligibility among the entire study population was 58%, with those living in states not contributing to the ADAP budget less likely to have a suppressed viral load (51% versus 59%). In adjusted analyses, this association was not statistically significant (conventional Cox regression-adjusted HR 1.02, 95% CI 0.88–1.18; propensity score-matched HR 1.13, 95% CI 0.93–1.36).

### Association between ADAP waiting lists and treatment outcomes

Among the overall study population (N = 8,874), ART initiation after six months was higher among those living in a state with an existing ADAP waiting list than those living in a state without a list (73% versus 55%). A similar pattern was observed in this overall population for one-year virologic suppression (71% versus 58%). In regression-adjusted analyses, the hazard ratio based on living in a waiting list state was 1.73 (95% CI 1.45–2.07) for ART initiation and 1.21 (95% CI 1.01–1.44) for virologic suppression (Table 4). After propensity score matching to improve exchangeability between groups, living in a waiting list state was no longer associated with delayed ART initiation (N = 620, HR 1.12, 95% CI 0.87–1.45) or virologic suppression (HR 1.05, 0.79–1.38). However, our analysis among IDU maintained the significant association between living in a waiting list state and ART initiation (HR 2.15, 95% CI 1.31–3.55).

**Table 4:** Association between living in an ADAP waiting list state and ART initiation and virologic suppression, U.S. NA-ACCORD, 2001–2009.

	Outcome: 6-month ART initiation		Outcome: 1-year virologic suppression	
	HR	95% CI	HR	95% CI
Overall				
Living in a waiting list state (vs. not living in a waiting list state)				
Crude (N = 8,874)	1.55	1.38–1.73	1.39	1.24–1.57
**Regression-adjusted (N = 8,874)**	**1.73**	**1.45–2.07**	**1.21**	**1.01–1.44**
**Propensity score-matched (N = 620)** *	**1.12**	**0.87–1.45**	**1.05**	**0.79–1.38**
Injection drug users only (N = 1,824)				
Living in a waiting list state (vs. not living in a waiting list state)				
Crude	1.59	1.19–2.11	1.49	1.10–2.03
**Regression-adjusted**	**2.15**	**1.31–3.55**	**1.30**	**0.80–2.09**

ART = antiretroviral therapy, CI = confidence interval, HR = hazard ratio.

All analyses use Cox proportional hazards regression.

* Hazard ratios obtained after 1∶3 matching (with replacement) 398 “exposed” to 222 “unexposed” individuals based on propensity of living in a waiting list state.

Both regression-adjusted and propensity-score matched analyses account for the following variables: age; sex; race/ethnicity; transmission risk; CD4+ count and viral load at eligibility; history of alcohol abuse, substance abuse, and mental disorders; year of eligibility; type of cohort; clinic-specific mechanisms to help obtain ART; state-level population density, % population of black race, % population below poverty line, median household income, and per capita Medicaid spending on HIV.

We performed a sensitivity analysis to examine whether the non-significant association was maintained when shortening the time to ART initiation to 3 months after eligibility instead of 6 months. Here, the HR was 1.44 (95% CI 1.06–1.97) (Table S2 of File S1). When we did not account for clinic-specific mechanisms to obtain treatment for patients in the propensity score model, the associations between living in a waiting list state and our outcomes of interest were greater and reached statistical significance (HR 1.93, 95% CI 1.49–2.51 for ART initiation, HR 1.29, 95% CI 1.02–1.63 for virologic suppression) compared with the base case scenario. Thus, additional follow-up time and confounder control seemed to attenuate the association between living in a waiting list state and ART initiation.

## Discussion

In this study of HIV-infected individuals in the United States who were newly clinically eligible to begin ART, we found that not having an additional state contribution to an ADAP's annual budget was associated with delayed ART initiation. This finding was robust to the type of statistical procedure used to account for known confounders, and furthermore was maintained when considering different assumptions, and when focusing on specific subpopulations, including those with a history of IDU.

Our findings are consistent with an ecologic analysis that suggested greater HIV inequities in some U.S. states as a result of lower state ADAP contributions [36]. Combining this information with our *a priori* hypothesis and the dose-response effect identified, we believe that we have described a plausible mechanism in delayed ART uptake. Several reports have noted the importance of state contributions to ADAP budgets [37], [38]. While discretionary federal funds to ADAP are proportionally allocated to states on the basis of HIV prevalence, in principle to guarantee distributional equity [39], this metric may not measure all aspects of need in individual states. The additional funding stream based on state general revenue may play a role in maintaining the core functions of the program or help to improve treatment uptake in the target population, such as the inadequately insured or the working poor. It could also result in more or better trained ADAP office staff to work with and follow up with clients or in better ancillary client services like adherence support.

We found that living in a state with an active ADAP waiting list was not associated with less timely ART initiation, and in fact, in some scenarios associated with more timely ART initiation. On the surface, this may seem paradoxical; we expected that living in a state with an ADAP waiting list would be associated with less timely ART initiation. However, this finding may reflect efforts at study sites contributing data to NA-ACCORD to get patients promptly treated when there is knowledge of existing structural barriers. For example, the more timely initiation related to waiting lists that we observed among IDU could reflect special efforts by sites to engage this high-need group into care, since it is known that IDU have lower levels of engagement in HIV care compared with other risk groups [25]. While we controlled for some clinic-level behaviors regarding assistance with procurement of ART, we may not have fully captured the scope of these efforts, since assessments were conducted retrospectively based on a survey of participating clinics. Furthermore, additional efforts occurring at state ADAP offices (e.g., efforts to help people sign up for pharmaceutical company prescription assistance programs when being placed on a waiting list, use of other cost-containment strategies when resources are low) were not assessed in our study. In our analysis, excluding clinic-based efforts from the propensity score model resulted in more pronounced and statistically significant associations between living in a waiting list state and ART initiation and virologic suppression, suggesting that we at least partially accounted for clinic-level factors related to ART initiation. Having additional information on the mechanisms that people use to access treatment would further inform this important data consideration.

We used propensity score matching methods to create comparable groups of “exposed” and “unexposed” individuals, capitalizing on the heterogeneity of policies across different states in the NA-ACCORD. This technique measures the “average treatment effect in the treated” population, which is different from the “average treatment effect” in the entire study population that conventional regression analyses assess. We can interpret our propensity-score matched estimates regarding state ADAP features as applicable to the subset of individuals with the same risk factor distribution as those living in those states with those features (i.e., no state contribution to the ADAP budget; presence of ADAP waiting lists) [40]. Thus, these findings may not necessarily apply to those with different risk factor distributions, or those who were not selected as a match. Nonetheless, when we ran conventional regression models that estimated effects among the entire study population, the results were generally similar to the propensity score-based results, lending further support to our conclusions.

We did not find significant associations between less generous ADAP features and less timely virologic suppression. One possibility for this is that the majority of HIV-infected individuals in our population were eventually treated (the percentage increasing to 65% overall after one year of eligibility), and once they began treatment, differences in the state ADAP features we examined may have played less of a role. In other words, the majority of people reached guideline-defined treatment goals, despite the delay in starting therapy that more limited state budgets may influence. This is encouraging, even though the additional efforts expended to procure treatment in light of these delays have costs.

Furthermore, we did not report on longer-term outcomes like sustained viral load suppression and mortality. Because our study is essentially an intent-to-treat analysis, we did not take into account changes in ADAP features over the course of an individuals' treatment trajectory. An analysis of time-updated ADAP changes could help to understand these processes better, especially considering the variability in coverage by some state ADAPs of medications for other health conditions relevant to HIV-infected individuals like hepatitis infection, cardiovascular disease, and mental health conditions [15], [41]–[43].

We originally hypothesized that our effect estimates would be greater among IDU owing to their increased needs with respect to care engagement and treatment initiation. While our data provide some evidence of this, the overall effects are not dramatically different from those overall, suggesting that on the whole, state-level differences in the ADAP features we examined may affect their target populations similarly with respect to ART initiation. However, it is possible that other differences in state ADAP formularies, such as coverage of hepatitis treatment or opioid dependency [23], [44], could influence outcomes more likely to affect IDU such as liver disease and drug overdose, and this is worth exploring.

Recent observational studies have taken other approaches to understand the influence of ADAP features, directly examining the benefits of ADAP enrollment itself on treatment utilization [42], [45], [46]. For example, the Women's Interagency HIV Study found increased use of ART among HIV-infected women enrolled in an ADAP versus those not enrolled, even after adjusting for insurance status [42]. A study from the 1917 Clinic in Alabama found that many ADAP enrollees, despite having ART available to them, still use ART suboptimally [45]. Because these studies focused on ADAP enrollment as an exposure itself, they examined pathways related to successful use of treatment as a consequence of enrollment. Our analysis complements these studies by providing information on earlier mechanisms that are a function of the state-related features of the ADAPs themselves, to address potential barriers to ADAP enrollment and therefore timely initiation of treatment. Our study is the largest to date examining the role of ADAP on treatment outcomes. Importantly, six of the ten states with the highest ADAP enrollments in the country were among the largest ten states represented in our study population (California, New York, Florida, Texas, Illinois, Pennsylvania) [18]. We also used consistent methods across states in our analysis, which would be more difficult to accomplish systematically using data from individual state programs [47]. However, our study has limitations. First, although NA-ACCORD sites are diverse and represent a variety of research settings [26], [48], many of the participating clinics are located at major academic centers, and therefore our inferences may be less generalizable to patients not seen at such clinics. However, we are reassured somewhat by the fact that many of these sites are responsible for the majority of HIV care in their respective catchment areas, and therefore are applicable to a large proportion of the general HIV-infected population. Second, because information on individual-level socioeconomic status or insurance status (including actual ADAP enrollment itself for each of the participants) was not available, our study population includes both people who are financially eligible for ADAP services (i.e., lower income) and people who may not qualify for assistance (i.e., higher income). Thus, we could not specifically study the subset of our population that was the true population at risk. Because of the ecologic nature of this analysis, the effects we estimated could be considered “contextual” effects, in that they apply to those living in the state during which a particular ADAP feature was in place, and not just those who were actually enrolled in an ADAP. Nonetheless, such contextual effects are useful since they suggest benefits from policies that go beyond the narrower population of ADAP enrollees.

Another limitation is that our exposures of interest were based on the results of annual surveys of state ADAP offices conducted by NASTAD over the study period and therefore are dependent on the quality of these findings. However, these results are publicly available and therefore allow for transparency should similar assessments be conducted by other investigators. Unmeasured confounding may have also affected our effect estimates. Both propensity score matching and conventional regression techniques are designed to account for observed confounders, but there may be other characteristics of patients, clinics, or the states themselves that we have not accounted for in our analysis. For example, we did not account for the diffusion of each state's ADAP program among its HIV-infected population, or more nuanced differences in state Medicaid eligibility or generosity beyond per capita HIV spending, which if important could lead to some bias in our conclusions. In sensitivity analyses, we controlled for state fixed effects to try to account for all of the unobserved characteristics of a particular state, but by doing so this technique may have over-adjusted for these effects, which may have been highly correlated with the exposure of interest.

Finally, the period of eligibility for this analysis ended in 2009, when at least two major changes occurred in the HIV epidemic in the United States: the adoption of clinical guidelines recommending starting treatment at a CD4+ count of 500 cells/uL or even higher [49], and a substantial rise in the number of people in the U.S. on ADAP waiting lists in 2010 and 2011, due to state-level economic crises [15], [18]. While better understanding of more recent changes is needed, our analysis nonetheless covered a significant portion of the history of the ADAP program. Future work should monitor ongoing changes to the healthcare funding landscape [50].

In conclusion, our study found an association between living in a state that does not provide an additional contribution to ADAP funding and delays in ART initiation. The importance of timely ART initiation when clinically indicated is well-established [17]. Many factors complicate the healthcare environment for people with HIV infection in the United States, including competition for resources as more people are tested and treated earlier [51], [52] and evolving trends in health insurance coverage [53], which will likely further change as Medicaid eligibility expands with the implementation of the Patient Protection and Affordable Care Act [54]–[56]. Because of these changes, more research on the impact of budgetary differences on the effectiveness of state ADAPs in providing timely therapies is clearly warranted, particularly for the groups that need this assistance the most. Such additional information may help ADAPs to better manage their resources and best serve the needs of their target populations.

## Supporting Information

File S1 Appendix. Table S1. Mechanisms at individual clinics in NA-ACCORD to assist patients in accessing prescription drugs in 2008 (N = 22). Table S2. Sensitivity analyses for association between state ADAP features at time of eligibility and outcomes.
(DOCX)
