# Supplementary material for: Association between U.S. State AIDS Drug Assistance Program (ADAP) Features and HIV Antiretroviral Therapy Initiation, 2001–2009
Source: PLoS One. 2013 Nov 18;8(11):e78952. doi: 10.1371/journal.pone.0078952 (PMC3832515; doi:10.1371/journal.pone.0078952)
Supplement: File S1 — Appendix. Table S1. Mechanisms at individual clinics in NA-ACCORD to assist patients in accessing prescription drugs in 2008 (N = 22). Table S2. Sensitivity analyses for association between state ADAP features at time of eligibility and outcomes. (DOCX) [file pone.0078952.s001.docx]

**APPENDIX**

*Clinic questionnaire.* We developed a questionnaire distributed to each clinical cohort that aimed to better characterize mechanisms in place at individual clinics to assist patients in accessing prescription drugs, in 2001 and 2008. The questionnaire, addressed to the medical director or other relevant authority with knowledge of this information, asked whether the clinic provided direct assistance with applying to the following services: Medicare Part D, Medicaid, ADAP, patient assistance programs from either individual drug companies or local pharmacies, or an AIDS clinical trial group. It also asked whether the clinic referred the patient to entities outside of the clinic for help to apply to such services. Results of this questionnaire for 2008 (N=22 clinics) are shown in Table S1.

For analysis purposes, we summarized these data by creating two scores, one for direct mechanisms and one for those referred to outside entities, by adding the dichotomous values for each of the individual mechanisms. For cohorts not surveyed, we used the median of the answers from the surveyed cohorts.

*Details of propensity score matching.* We used tools in the MatchIt package [1] in R 2.15.0 (R Foundation for Statistical Computing, Vienna, Austria) to perform propensity score matching. We estimated the predicted probability of living in a state with each feature using logistic regression, conditioning on the following individual- and clinic-level variables that might confound the relationship between the exposure and the outcomes of interest at the time of eligibility: age (continuous), race/ethnicity (black, Hispanic, and white or other), sex and transmission risk (men who have sex with men, male IDU, female IDU, male other risk, female other risk), CD4+ count (<200 cells/uL, 200-349 cells/uL, 350+ cells/uL), viral load (undetectable, 500-9,999 copies/mL, 10,000-99,999 copies/mL, 100,000 copies/mL or more, missing), number of psychosocial barriers (ordinal, 0 through 3), calendar year (ordinal, 2001 through 2009), and clinic-specific mechanisms for obtaining prescription drugs (continuous, and grouped into 2 variables: internal and external/referral-based). We also included the following state-level variables, parameterized into quartiles and analyzed as indicator variables: population density, percent of population that is black, median household income, percent of population below the FPL, and Medicaid spending on HIV per capita.

To minimize differences between the “exposed” and “unexposed”, we matched exposed individuals to comparable unexposed individuals based on the predicted probabilities, or propensity scores. We evaluated different matching methods both quantitatively based on the estimation of “standardized bias” and qualitatively by examining the distribution of propensity scores across exposed and unexposed individuals. Standardized bias is the “difference in means of each covariate [before and after matching], divided by the standard deviation in the full treated group”.[2] A standardized bias value less than 0.25 has been described as “trustworthy” for matching purposes.[3] Parameters that we evaluated for different nearest neighbor matching methods included the number of unexposed individuals chosen (up to 4) and whether matching with replacement is used. Matching with replacement means that exposed individuals can be matched to more than one unexposed individual if necessary. In general, we found the lowest average standardized bias across all variables through the use of 1:3 nearest neighbor matching (exposed:unexposed) with replacement.

We graphically assessed whether there was sufficient overlap in the distributions between exposed and unexposed individuals to have comparable populations by examining propensity score distributions. This is known in the literature as “common support”, an essential feature of exchangeability. For the state contribution to the ADAP budget variable, there was common support without having to alter the matching strategy.[4] For the ADAP waiting list variable, a proportion of the exposed individuals (i.e., individuals living in a state with a waiting list at the time of ART eligibility) did not have comparable unexposed individuals. To maintain common support, we excluded 43 out of 441 exposed individuals from analysis (9.8%).[5] A drawback of this approach is that the resulting estimated only describes the subpopulation of exposed individuals whose propensities are overall similar to those of the unexposed individuals.[6]

*Sensitivity analyses.* We performed several sensitivity analyses to examine assumptions about the relationship between state ADAP features and the outcomes of interest (Table S2). Methodological alternatives that we examined included additional control for state fixed effects (to account for any unobserved state effects), not accounting for clinic-specific mechanisms to procure ART treatment, using inverse probability weighting to account for confounding instead of propensity score matching, and additional control for state level of insurance coverage (based on time-varying data obtained from the Current Population Survey). We also lagged exposures by 6 months in case the policies had a delayed effect on changing ART initiation patterns in this population. Finally, we explored effect estimates in several subgroups, including confirmed newly eligible individuals (i.e., those with a prior recorded CD4+ count >350 cells/uL), individuals whose state of residence was recorded (versus presumed), and injection drug users defined using a more sensitive definition (i.e., documented history of IDU only).

**APPENDIX REFERENCES**

1. Ho D, Imai K, King G, Stuart E (2007). Matching as nonparametric preprocessing for reducing model dependence in parametric causal inference. Pol Analysis 15:199-236.

2. Stuart EA (2010). Matching methods for causal inference: A review and a look forward. Stat Sci 25(1): 1-21.

3. Rubin DB (2001). Using propensity scores to help design observational studies: Application to the tobacco litigation. Health Serv Outcomes Res Methodol 2:169-188.

4. Imbens GW (2004). Nonparametric estimation of average treatment effects under exogeneity: A review. Rev Econ Stat 86(1): 4-29.

5 Schafer JL, Kang J (2008). Average causal effects from nonrandomized studies: a practical guide and simulated example. Psychol Methods 13(4): 279-313.

6 Crump RK, Hotz VJ, Imbens GW, Mitnik OA (2009). Dealing with limited overlap in estimation of average treatment effects. Biometrika 96(1): 187-199

**Table S1. Mechanisms at individual clinics in NA-ACCORD to assist patients in accessing prescription drugs in 2008 (N=22).**

|  | **N** | **%** |
| --- | --- | --- |
| Referral to: |  |  |
| Outside (non-clinic) staff within organization to provide help with obtaining drug coverage | 8 | 36 |
| Outside organizations that provide help with obtaining drug coverage | 12 | 55 |
| Patient assistance programs from individual drug companies | 14 | 64 |
| Direct assistance with applying to: |  |  |
| Medicare Part D | 19 | 86 |
| State Medicaid program | 20 | 91 |
| State AIDS Drug Assistance Program (ADAP) | 21 | 96 |
| Patient assistance programs from individual drug companies | 19 | 86 |
| Patient assistance program from local pharmacy | 8 | 36 |
| AIDS Clinical Trial Group | 15 | 68 |
| Other federal or state program | 2 | 9 |
| Other local program | 2 | 9 |
| Other clinical trials | 3 | 14 |

The median number of procedures for referrals for the study population was 4 (interquartile range [IQR] 3-5) and for direct assistance was 0 (IQR 0-1).

Findings were similar for mechanisms existing in 2001.

**Table S2. Sensitivity analyses for association between state ADAP features at time of eligibility and outcomes.**

|  | **N,** | **N,** | **Outcome: 6-month ART initiation** | | **Outcome: 1-year virologic suppression** | |
| --- | --- | --- | --- | --- | --- | --- |
|  | **unexposed** | **exposed** | **HR** | **95% CI** | **HR** | **95% CI** |
| **No state contribution to the ADAP budget (versus any contribution)** | | | | | | |
| **Base case** | **399** | **683** | **0.73** | **0.60-0.88** | **1.13** | **0.93-1.36** |
| Sensitivity analyses: Methodological | | | | | | |
| Control for state fixed effects | 399 | 683 | 0.73 | 0.59-0.90 | 1.06 | 0.87-1.30 |
| Do not account for clinic-specific mechanisms in PS model | 399 | 683 | 0.79 | 0.67-0.94 | 1.07 | 0.90-1.26 |
| Use weighting instead of PS matching* | 8191 | 683 | 0.84 | 0.69-1.03 | 1.06 | 0.87-1.29 |
| Control for state insurance coverage | 279 | 683 | 0.77 | 0.62-0.97 | 0.90 | 0.74-1.11 |
| Sensitivity analyses: Modify exposure definition | | | | | | |
| Based on 6-month lagged exposure | 343 | 689 | 0.91 | 0.73-1.14 | 1.41 | 1.14-1.74 |
| Sensitivity analyses: Subgroups | |  |  |  |  |  |
| Confirmed newly treatment-eligible individuals | 200 | 390 | 0.88 | 0.63-1.23 | 1.16 | 0.84-1.58 |
| Confirmed state of residence (vs. presumed) | 244 | 491 | 0.93 | 0.72-1.21 | 1.18 | 0.91-1.52 |
| Confirmed IDU (vs. presumed)** | 1109 | 224 | 0.62 | 0.41-0.94 | 1.11 | 0.75-1.64 |
| **Living in a waiting list state (versus not living in a waiting list state)** | | | | | | |
| **Base case** | **222** | **398** | **1.12** | **0.87-1.45** | **1.05** | **0.79-1.38** |
| Sensitivity analyses: Methodological | | | | | | |
| Control for state fixed effects | 222 | 398 | 1.13 | 0.89-1.44 | 1.04 | 0.81-1.34 |
| Do not account for clinic-specific mechanisms in PS model | 222 | 398 | 1.93 | 1.49-2.51 | 1.29 | 1.02-1.63 |
| Use weighting instead of PS matching* | 8433 | 441 | 1.41 | 1.09-1.81 | 1.05 | 0.83-1.34 |
| Control for state insurance coverage | 199 | 414 | 1.18 | 0.91-1.52 | 0.91 | 0.70-1.18 |
| Sensitivity analyses: Modify exposure or outcome definition | | | | | | |
| Based on 6-month lagged exposure | 243 | 204 | 0.94 | 0.75-1.17 | 1.06 | 0.85-1.34 |
| Based on 3-month ART initiation outcome | 222 | 398 | 1.44 | 1.06-1.97 | - | - |
| Sensitivity analyses: Subgroups | | | | | | |
| Confirmed newly treatment-eligible individuals | 89 | 92 | 1.73 | 1.11-2.72 | 1.10 | 0.71-1.68 |
| Confirmed state of residence (vs. presumed) | 147 | 232 | 1.24 | 0.91-1.67 | 0.96 | 0.71-1.30 |
| Confirmed IDU (vs. presumed)** | 1290 | 43 | 2.85 | 1.44-5.65 | 1.92 | 1.01-3.65 |

ADAP = AIDS Drug Assistance Program, ART = antiretroviral therapy, CI = confidence interval, HR = hazard ratio, IDU = injection drug user, PS = propensity score.

Hazard ratios obtained after 1:3 matching “exposed” individuals to “unexposed” individuals based on propensity of exposure to each feature, with replacement, except as noted: *inverse probability weighting and **conventional regression analysis. All analyses account for the following variables: age; sex; race/ethnicity; transmission risk; CD4+ count and viral load at eligibility; history of alcohol abuse, substance abuse, and mental disorders; year of eligibility; type of cohort; clinic-specific mechanisms to help obtain ART; state-level population density, % population of black race, % population below poverty line, median household income, and per capita HIV Medicaid spending.
